# Parasagittal dural space and cerebrospinal fluid (CSF) flow across the lifespan in healthy adults

**DOI:** 10.1186/s12987-022-00320-4

**Published:** 2022-03-21

**Authors:** Kilian Hett, Colin D. McKnight, Jarrod J. Eisma, Jason Elenberger, Jennifer S. Lindsey, Ciaran M. Considine, Daniel O. Claassen, Manus J. Donahue

**Affiliations:** 1 Department of Neurology, Vanderbilt University Medical Center, Nashville, TN USA; 2 Department of Radiology and Radiological Sciences, Vanderbilt University Medical Center, Nashville, TN USA; 3 Department of Psychiatry and Behavioral Sciences, Vanderbilt University Medical Center, Nashville, TN USA; 4 Division of Behavioral and Cognitive Neurology, Village at Vanderbilt, 1500 21st Avenue South, Nashville, TN 37212 USA

**Keywords:** Parasagittal dura space, Cerebral aqueduct, Glymphatic, Cerebrospinal fluid, Aging

## Abstract

**Background:**

Recent studies have suggested alternative cerebrospinal fluid (CSF) clearance pathways for brain parenchymal metabolic waste products. One fundamental but relatively under-explored component of these pathways is the anatomic region surrounding the superior sagittal sinus, which has been shown to have relevance to trans-arachnoid molecular passage. This so-called parasagittal dural (PSD) space may play a physiologically significant role as a distal intracranial component of the human glymphatic circuit, yet fundamental gaps persist in our knowledge of how this space changes with normal aging and intracranial bulk fluid transport.

**Methods:**

We re-parameterized MRI methods to assess CSF circulation in humans using high resolution imaging of the PSD space and phase contrast measures of flow through the cerebral aqueduct to test the hypotheses that volumetric measures of PSD space (1) are directly related to CSF flow (mL/s) through the cerebral aqueduct, and (2) increase with age. Multi-modal 3-Tesla MRI was applied in healthy participants (n = 62; age range = 20–83 years) across the adult lifespan whereby phase contrast assessments of CSF flow through the aqueduct were paired with non-contrasted *T*_1_-weighted and *T*_2_-weighted MRI for PSD volumetry. PSD volume was extracted using a recently validated neural networks algorithm. Non-parametric regression models were applied to evaluate how PSD volume related to tissue volume and age cross-sectionally, and separately how PSD volume related to CSF flow (significance criteria: two-sided *p* < 0.05).

**Results:**

A significant PSD volume enlargement in relation to normal aging (*p* < 0.001, Spearman’s-$\rho$ = 0.6), CSF volume (*p* < 0.001, Spearman’s-$\rho$ = 0.6) and maximum CSF flow through the aqueduct of Sylvius (anterograde and retrograde, *p* < 0.001) were observed. The elevation in PSD volume was not significantly related to gray or white matter tissue volumes. Findings are consistent with PSD volume increasing with age and bulk CSF flow.

**Conclusions:**

Findings highlight the feasibility of quantifying PSD volume non-invasively in vivo in humans using machine learning and non-contrast MRI. Additionally, findings demonstrate that PSD volume increases with age and relates to CSF volume and bi-directional flow. Values reported should provide useful normative ranges for how PSD volume adjusts with age, which will serve as a necessary pre-requisite for comparisons to persons with neurodegenerative disorders.

**Supplementary Information:**

The online version contains supplementary material available at 10.1186/s12987-022-00320-4.

## Introduction

Classically, cerebrospinal fluid (CSF) production is recognized to occur in the choroid plexus (ChP) complexes, after which the fluid circulates through the ventricular system and around the brain and spinal cord with subsequent resorption mediated by dural venous sinus arachnoid granulations. However, there is emerging evidence that CSF clearance may also occur in the regions surrounding the dural sinuses [1]. Support for this possibility comes from intrathecal gadolinium contrast administration in 18 human subjects, whereby contrast was noted to concentrate in the CSF spaces near the vertex [2]. With passage of time, contrast progressively accumulated within the tissue surrounding the superior sagittal sinus, or the parasagittal dural (PSD) space. These findings suggest that trans-arachnoid molecular passage may occur in spaces surrounding the superior sagittal sinus and highlights that the PSD morphology may have relevance for effective fluid transport. Despite this, there is limited knowledge of how variance in PSD space relates to aging, brain health, and intracranial CSF flow.

The primary source of CSF production is the ChP, which is estimated to produce approximately 500 mL of CSF daily at a rate of approximately 20–25 mL CSF per hour in adult humans [3]. While most of the ChP tissue resides within the atria of the lateral ventricles, there is choroidal tissue throughout the ventricular system in rough proportion to the overall size of the ventricular components. CSF produced in the lateral and third ventricles traverses the cerebral aqueduct (i.e., aqueduct of Sylvius) en route to the 4th ventricle and on to the more diffuse subarachnoid space [4, 5]. As the cerebral aqueduct represents the sole pathway for CSF efflux from the lateral and third ventricles, measurement of flow in this region offers an opportunity to quantify fluid production by the third and lateral ventricles, which comprise the largest ChP complexes. To measure CSF efflux in the cerebral aqueduct, magnetic resonance (MR) phase contrast sequences, traditionally sensitive to arterial or venous flow, have been re-parameterized to enable quantitative CSF flow assessments in units of mL CSF per second, primarily by pairing with cardiac phase and reducing the velocity encoding gradient to coincide with maximum CSF flow velocities (typically 10–15 cm/s). [6]. Aberrant phase contrast flow parameters have long been associated with idiopathic normal pressure hydrocephalus [7]. CSF flow through the cerebral aqueduct is also dependent on cardiac phase and has been shown to be highest in older adults and in males vs. females [8]. These findings are consistent with previous studies that have shown age, and sex dependencies of CSF on tissue volume [9, 10], and suggest that CSF flow profiles may relate to total CSF volume.

The logical extension of this work, particularly given the increased attention to the role of the PSD in effective fluid transport from the subarachnoid space, is to understand how such CSF flow parameters relate to quantitative estimates of the PSD volume. Here, we applied deep learning algorithms to non-contrasted head MR images in healthy adults across the lifespan, for the first time, to test the hypotheses that increases in PSD volume parallel changes in CSF flow through the cerebral aqueduct, and additionally that PSD volume increases with advancing age. Findings are discussed in the context of the growing literature on PSD morphology and CSF clearance.

## Materials and methods

### Participants

All participants provided informed, written consent in accordance with the local institutional review board (IRB) and consistent with the Declaration of Helsinki and its amendments. All participants were scanned between January 2020 and September 2021 at Vanderbilt University Medical Center. Inclusion criteria for healthy control participants: age = 20–83 years, no history of cerebrovascular disease, anemia, psychosis, or neurological disorder including but not limited to prior overt stroke, sickle cell anemia, schizophrenia, bipolar disorder, existing neurodegenerative disorder, or multiple sclerosis. Presence of non-specific white matter lesions was not an exclusion criterion, as these lesions are extremely prevalent with aging, and we sought our cohort to be generalizable and representative. Clinical history was reviewed by a board-certified Neurologist (DOC; experience = 14 years) and anatomical imaging and angiography by a board-certified neuroradiologist (CDM; experience = 12 years) to ensure that inclusion criteria were met.

### Acquisition

All participants were scanned at 3.0 Tesla (Philips Healthcare, Best, The Netherlands) using body coil radiofrequency transmission and phased array 32-channel reception. The scan protocol included standard anatomical imaging consisting of 3D *T*_1_-weighted magnetization-prepared-rapid-gradient-echo (MPRAGE) (echo time = 8.1 ms, repetition time = 3.7 ms, flip angle = 8°, resolution = 1 × 1 × 1 mm), 3D *T*_2_-weighted volume isotropic-turbo-spin-echo-acquisition (VISTA) (echo time = 0.31 ms, repetition time = 2.7 ms, and spatial resolution = 0.78 × 0.78 × 0.78 mm), 2D *T*_2_-weighted FLAIR (echo time = 120 ms, repetition time = 11,000 ms, spatial resolution = 1 × 1 × 4 mm), 3D time-of-flight magnetic resonance angiography (echo time = 3.45 ms, repetition time = 23 ms, spatial resolution = 0.39 × 0.39 × 1.4 mm), and diffusion weighted imaging (DWI) (echo time = 83 ms, repetition time = 2923, b-value = 1000 s/mm^2^; spatial resolution = 1.8 × 1.8 × 4 mm). These scans were primarily used for confirming healthy status and ensuring inclusion criteria; *T*_1_-weighted and *T*_2_-weighted scans were additionally used for brain parenchymal and PSD volume segmentation as described below.

CSF movement was recorded within the aqueduct of Sylvius. To achieve this, four MR-compatible ECG electrodes were placed on the chest to enable retrospective cardiac phase correction. A single slice orthogonal to the aqueduct of Sylvius was placed above the location of the 4th ventricle, where the aqueduct is bound by the tectum posteriorly and mid-brain anteriorly. A velocity encoding gradient of 12 cm/s was applied, and measurements were acquired for five minutes, after which 12 cardiac phases were time-averaged to generate the mean CSF flow profile over the cardiac cycle.

### Analysis

**Fig. 1:**
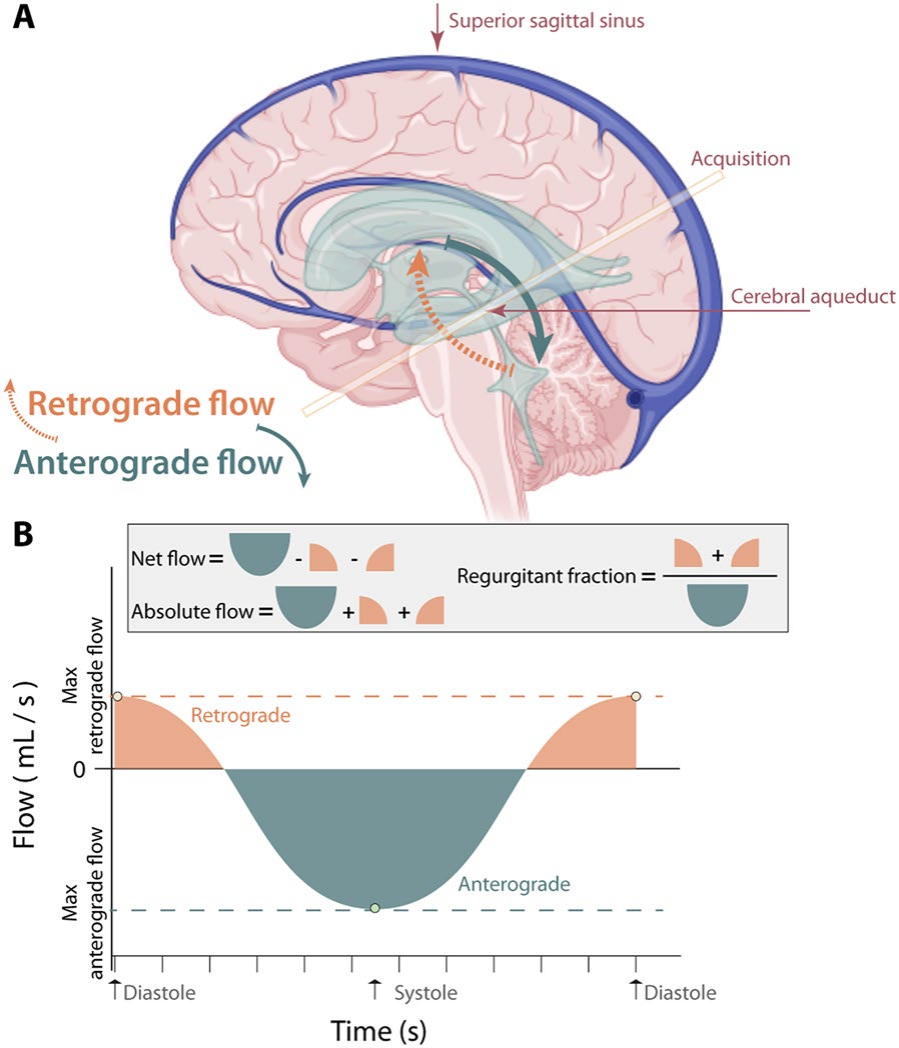
Cerebrospinal fluid (CSF) flow. **A** Schematic showing the superior sagittal sinus, which is surrounded by the parasagittal dural space, as well as the bulk CSF flow pathway (light green). Here, anterograde flow (mL CSF per unit time) is defined as flow in the cranial-to-caudal direction down the aqueduct, which occurs during systole; retrograde flow is defined as flow in the caudal-to-cranial direction, which occurs during diastole. **B** A simulated CSF flow time course with terms used in subsequent analyses defined

*CSF flow* The directionality of CSF flow was defined as anterograde (from cranial to caudal) or retrograde (from caudal to cranial), whereby anterograde flow occurs primarily during systole and retrograde flow occurs primarily during diastole (Fig. 1). To estimate CSF flow in the aqueduct of Sylvius, we followed the acquisition protocol proposed in [6]. A mask of the aqueduct of Sylvius was generated from the magnitude data in the phase-contrast acquisition. Next, the mask was applied to the modulus of the phase-contrast readout to obtain the acquired phase in the moving CSF in the aqueduct of Sylvius over the duration of the entire cardiac cycle. Using the standard formula for the approximation of flow velocity from phase [11], mean volumetric flow (mL/s) was calculated by multiplying the mean velocity (cm/s) by the area of the aqueduct of Sylvius mask (cm^2^) and converting cubic millimeters to milliliters (1 cm^3^ = 1 mL). The mean volumetric flow of CSF through the aqueduct of Sylvius was calculated during 12 prescribed phases of the cardiac cycle which leads to computation of the following flow parameters: the maximum anterograde flow, maximum retrograde flow, net flow, absolute flow, and regurgitation fraction (Fig. 1).

*Anatomical characteristics* All brain volumes were calculated using the *T*_1_-weighted acquisitions. First, intracranial volume (ICV) was estimated using the brain mask computed by the advanced normalization tools (ANTs) package [12], which utilized the MNI ICBM-152 version as a template [13]. CSF, gray matter (GM), and white matter (WM) volumes were calculated using the Atropos method [14].

*Parasagittal dural (PSD) space quantification* PSD volumes were computed using a semi-supervised segmentation method based on a combination of a fully connected neural network (F-CNN) and voxel clustering based on a gaussian mixture model (GMM) to label voxels as PSD or sagittal sinus based on their *T*_2_-weighted MRI signal intensities. The first step of our method used an F-CNN to extract parasagittal space; this deep-learning model was trained using 20 *T*_2_-weighted MRI scans, as we found *T*_2_-weighted contrast to provides the best contrast to the adjacent subarachnoid space (relative to proton density or *T*_1_-weighted contrast). This deep-learning model aims to estimate a binary mask of parasagittal space which includes both the PSD, superior sagittal sinus, and contributing veins in the region of the PSD. Once the parasagittal space mask was computed, voxels belonging to the parasagittal mask were labeled as PSD, or superior sagittal sinus/contributing veins.

To achieve this, bias field inhomogeneity was reduced using N4 inhomogeneity correction [15]. Next, in order to reduce anatomical variability, all *T*_2_-weighted MRI images were aligned to the MNI template [13] using non-linear registration computed with ANTs [12] with a control spacing point set to 2 mm. This value provides equipoise between the robustness of the registration (i.e., limitation of eventual registration artifacts) and increase of inter-subject similarity of the PSD. The PSD volumes are obtained using a semi-supervised machine learning method as described below.

First, a board-certified neuroradiologist (CDM) manually segmented the parasagittal space of 20 *T*_2_-weighted scans. This region of interest contains the superior sagittal sinus volume and PSD space volume along the sinus. Next, manual segmentation maps were used to train an automatic segmentation method based on an F-CNN using a U-Net architecture [16]. U-Net architecture was chosen for its good performance with medical image segmentation task and its ability to accommodate limited training data sizes. In total, 180 overlapping patches were used to reconstruct the parasagittal space. Finally, a gaussian mixture model was fit, within the estimated parasagittal mask, using an a posteriori probability maximization strategy of the *T*_2_-weighted MRI signal distribution using Bayes framework.

Thus, the final maps provide labels for voxels belonging to the PSD space (hyperintense on the *T*_2_-weighted scans) and venous structures including the superior sagittal sinus and contributing veins (hypointense in the *T*_2_-weighted scans). Resulting segmentation maps were transformed to native space using the inverse transform. Therefore, all subsequent analyses were performed in the native space of the *T*_2_-weighted scans. All segmentation maps (PSD and tissue masks) were visually inspected and validated by a neuroradiologist (CDM).

### Statistical analysis

To assess the validity of our hypotheses, we used a generalized linear model (GLM). We defined three multivariate models to assess three different hypotheses.

1. *Demographics and intracranial cavity volume (ICV)* we defined a model with PSD and maximum anterograde or retrograde flow as separate dependent variables and age, sex, and intracranial volume as independent variables.
2. *Demographics and tissue volumes (GM, WM, and CSF)* we defined a model with PSD and maximum anterograde or retrograde flow as separate dependent variables and age, sex, CSF, GM, and WM volumes as independent variables.
3. *PSD volume and CSF flow* we defined a model with PSD volume as the dependent variable and the CSF flow characteristics including maximum anterograde flow, maximum retrograde flow, net flow (i.e., difference between net anterograde and retrograde flow), absolute flow (i.e., sum of total anterograde and retrograde flow per cardiac cycle), or regurgitant fraction (i.e., retrograde to anterograde flow ratio), as well as age, and sex as independent variables.

In addition to these linear models, for completeness we also assessed the correlation of each pair of features using the Spearman’s rank correlation coefficient. This was evaluated using analysis of variance (ANOVA) and corrected for multiple comparison using false discovery rate (FDR) [17]. Demographics data also have been categorized in three age groups, young (aged from 20 to 39 years old), middle (aged from 40 to 59 years old), and older (aged from 60 to 83 years old). Kruskal–Wallis non-parametric test has been performed to assess significant differences among the different age-groups. Confidence intervals of each linear regression analysis have been calculated using the Wald method. All reported *p*-values are reported as raw and corrected with FDR with significance fixed to 0.05. All analyses were performed in Matlab (Mathworks, Natick, MA) using the statistical toolbox.

## Results

### Demographics

A summary of participant demographics is provided in Table 1. In total, 62 participants were included, with an age ranging from 20 to 83 years inclusive. All participants met neurological and radiological inclusion criteria as defined in the “Materials and methods”.

**Table 1:** Demographic description of the cohort

	Age = 20–39 years	Age = 40–59 years	Age = 60–83 years	Total Age = 20–83 years	p
Age in years ($\sigma$)	27.5 (5.6)	51.3 (6.5)	70.2 (5.9)	50.4 (18.0)	–
Number of scans	20	22	23	65	–
Sex: Female/Male	15/5	12/10	15/8	42/23	0.3
ICV cm^3^ ($\sigma$)	1340 (101)	1407 (143)	1360 (135)	1371 (130)	0.3
CSF cm^3^ ($\sigma$)	239 (31)	279 (50)	318 (44)	280 (53)	< 0.01
GM cm^3^ ($\sigma$)	691 (63)	690 (63)	645 (55)	675 (59)	< 0.01
WM cm^3^ ($\sigma$)	409 (22)	438 (55)	396 (54)	415 (52)	0.06
Maximum anterograde flow mL/s ($\sigma$)	0.08 (0.03)	0.08 (0.04)	0.13 (0.07)	0.10 (0.06)	0.04
Maximum retrograde flow mL/s ($\sigma$)	− 0.10 (0.04)	− 0.11 (0.05)	− 0.16 (0.07)	− 0.12 (0.06)	< 0.01
PSD volume cm^3^ ($\sigma$)	7.05 (1.34)	8.31 (2.23)	9.66 (2.06)	8.36 (2.17)	< 0.01

Significance was evaluated using a Kruskal–Wallis’s test. *ICV* intra-cranial volume, *CSF* cerebrospinal fluid, *GM* gray matter, *WM* white matter, *PSD* parasagittal dura, Retrograde and anterograde designated the maximum flow in each direction (i.e., 3rd and lateral ventricles to 4th ventricles, and inversely)

### Volumetric results

No significant difference in ICV over the different age groups of 20–39 years, 40–59 years, or 60–83 years (mean ICV equal to 1371cm^3^, $\upsigma$ = 130cm^3^) or sex was observed. The analysis of the relationship between brain tissue volume and age indicates that GM significantly declines with age with p-value < 0.001 and $\uprho$=0.79, while no significant relationship was observed for WM using a linear model. Alternatively, CSF volumes significantly increased with age (p-value < 0.001 and $\uprho$ = 0.79; see Table 1 and Additional file 1: Table S1).

**Fig. 2:**
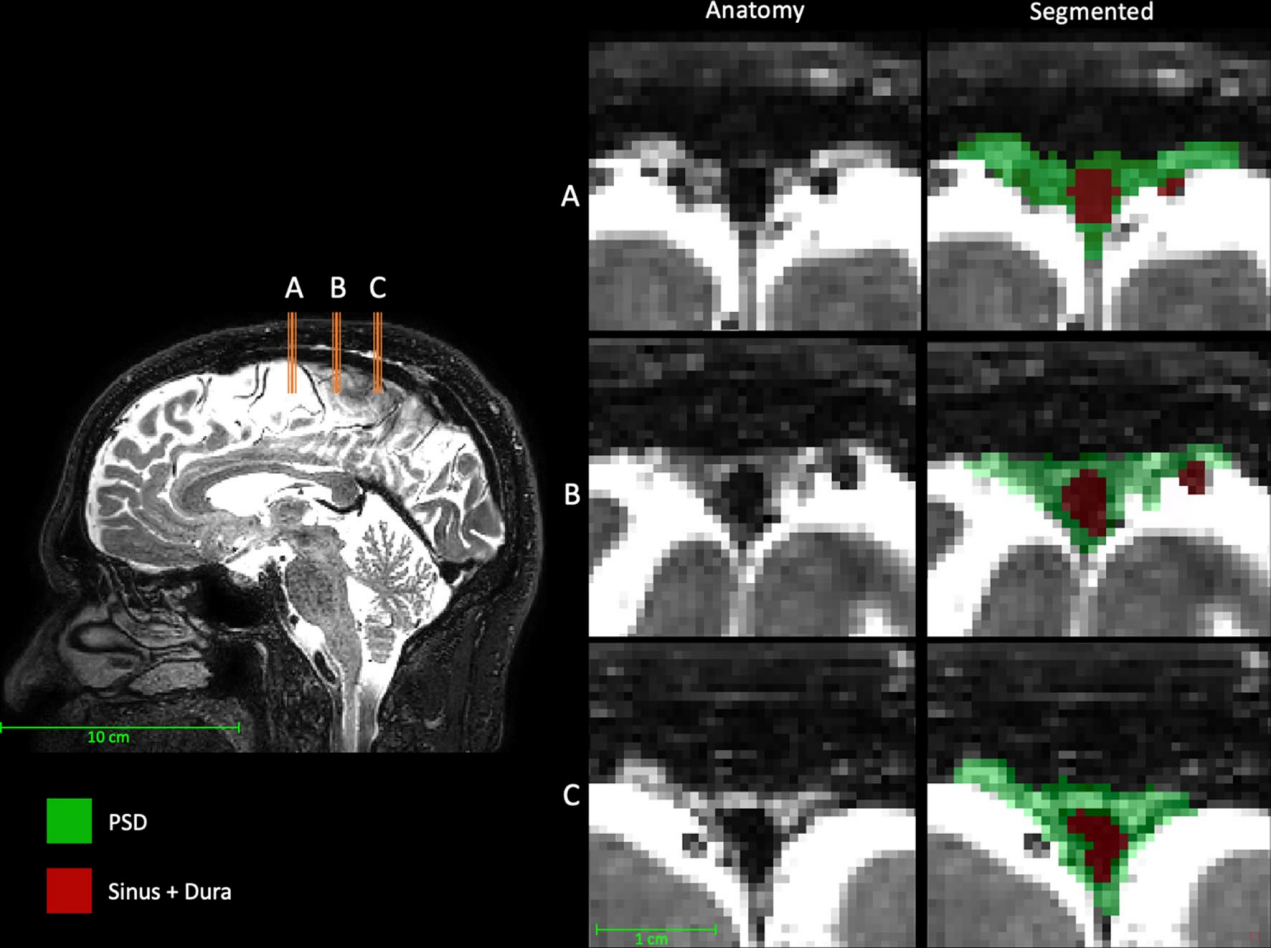
Parasagittal dura space (PSD) definition using *T*_2_-weighted MRI. Three coronal slices are taken from the posterior aspect of the frontal lobe to the medial aspect of the parietal lobe. In this figure the PSD appears in green, the remainder of the parasagittal space appears in red (i.e., sagittal sinus, and afferent veins)

**Fig. 3:**
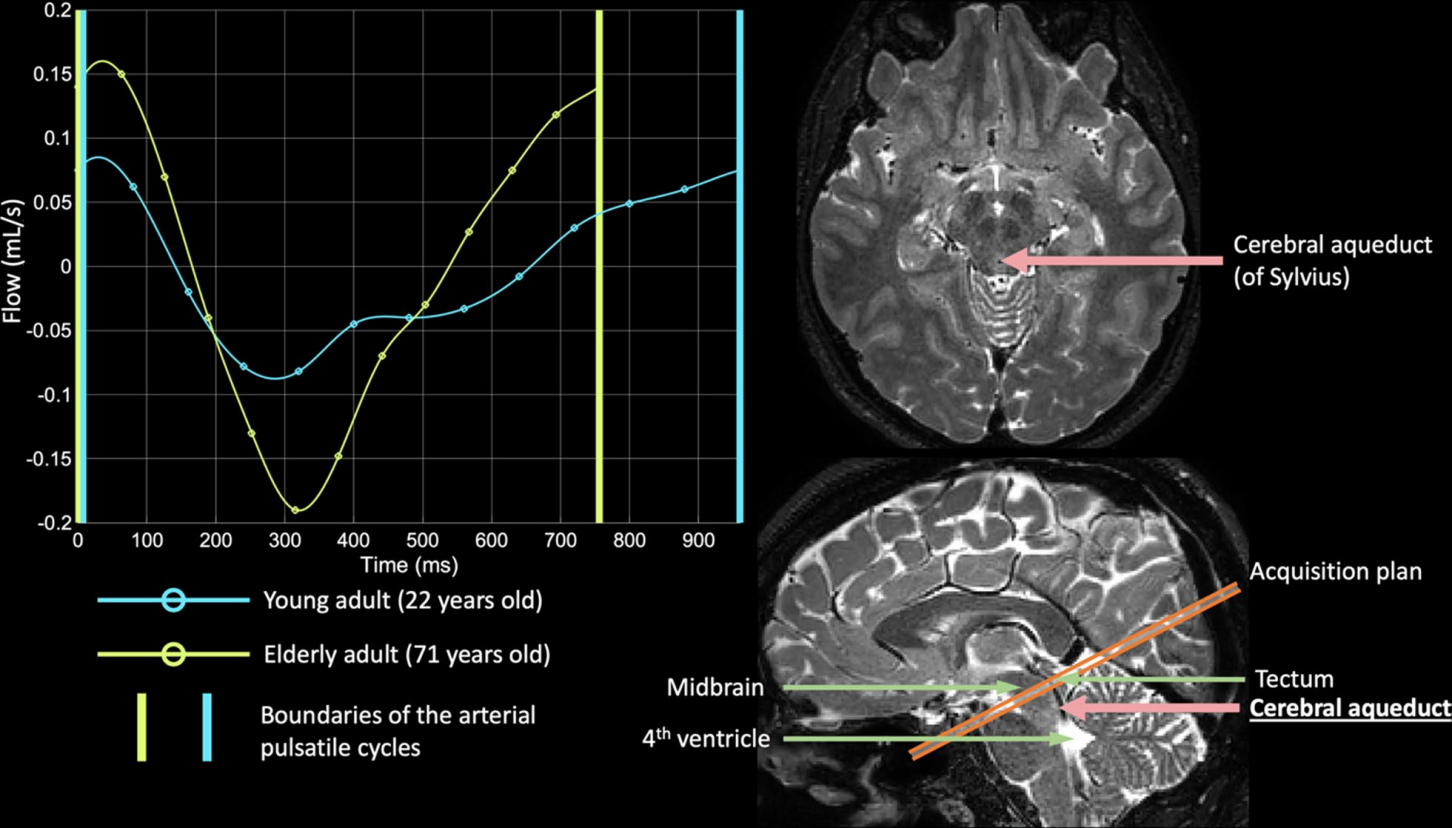
Illustration of cerebral aqueduct phase contrast flow acquisition. On the left, two curves representing the recorded CSF flow through the aqueduct of Sylvius over one arterial pulsatile cycle for a young adult (age = 22 years) and an older adult (age = 71 years). On the right, oblique axial and sagittal slices of *T*_2_-weighted MRI indicating the localization of the aqueduct of Sylvius. Due to high CSF flow through the aqueduct of Sylvius, the MR signal is dephased and appears hypointense on 3D T_2_-weighted MRI

**Fig. 4:**
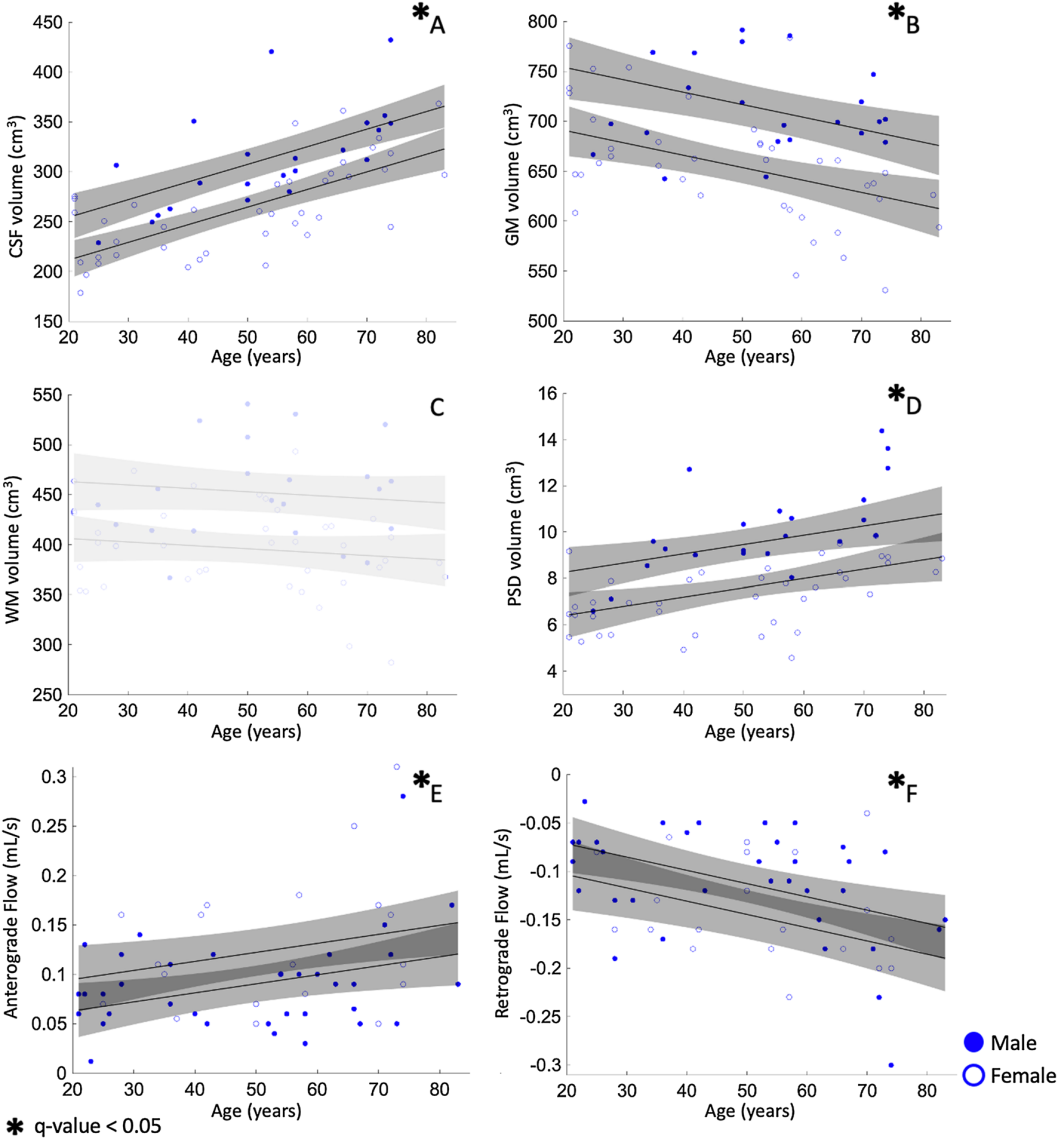
Relationship of age with different anatomical characteristics for (**A**) cerebrospinal fluid (CSF), (**B**) gray matter (GM), (**C**) white matter (WM), (**D**) parasagittal dural (PSD) volumes, (**E**) retrograde flow, and (**F**) anterograde flow in the cerebral aqueduct. As expected, data show a significant correlation of GM and CSF volume with age. In addition, this study shows a novel significant relationship of normal aging with PSD volume, and CSF flow in the cerebral aqueduct. Non-significant trends appear in light gray shade; significant trends appear in dark gray shade. Gray area corresponds to 95 percent confidence intervals estimated using the Wald method. Relationships that do not reach the significant threshold after multiple comparison correction appear in light gray shade; significant relationships are denoted with an asterisk and with confidence intervals appearing in dark gray shade

**Fig. 5:**
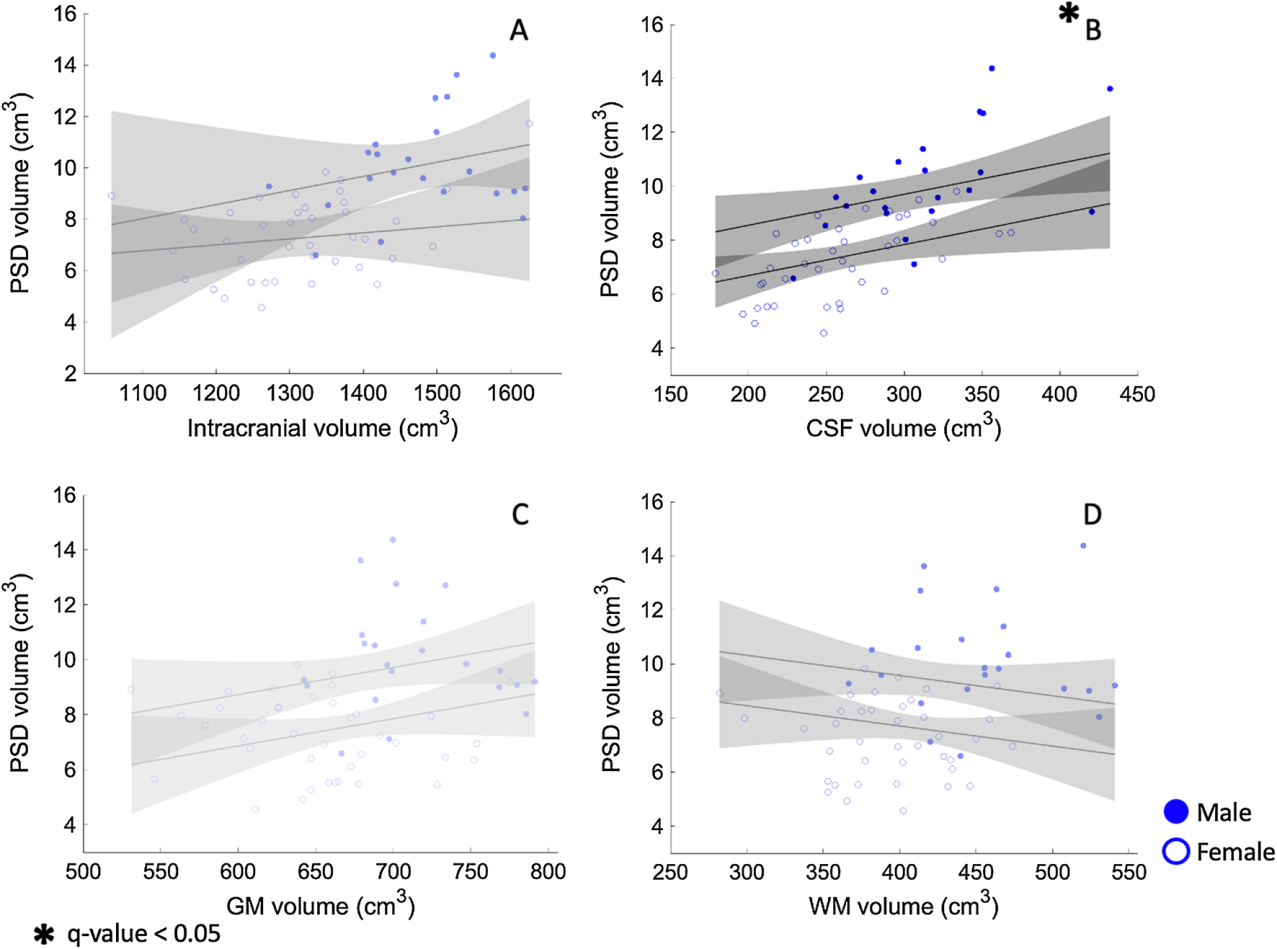
Correlation of parasagittal dural (PSD) and diverse anatomical measure. PSD does not show a significant relationship with total gray matter (GM) or white matter (WM). Analysis shows a significant relationship of PSD and cerebrospinal fluid (CSF) volume (p-value = 0.02, q-value = 0.04). Moreover, analysis shows a non-significant relationship with intracranial volume (ICV) when controlled for sex dependency. Gray area corresponds to 95 percent confidence interval estimated using the Wald method. Relationships *that do not reach* the *significant threshold after multiple comparison correction* appear in light gray shade; significant relationships *are denoted with an asterisk and with confidence intervals* appear*ing* in dark gray shade

**Fig. 6:**
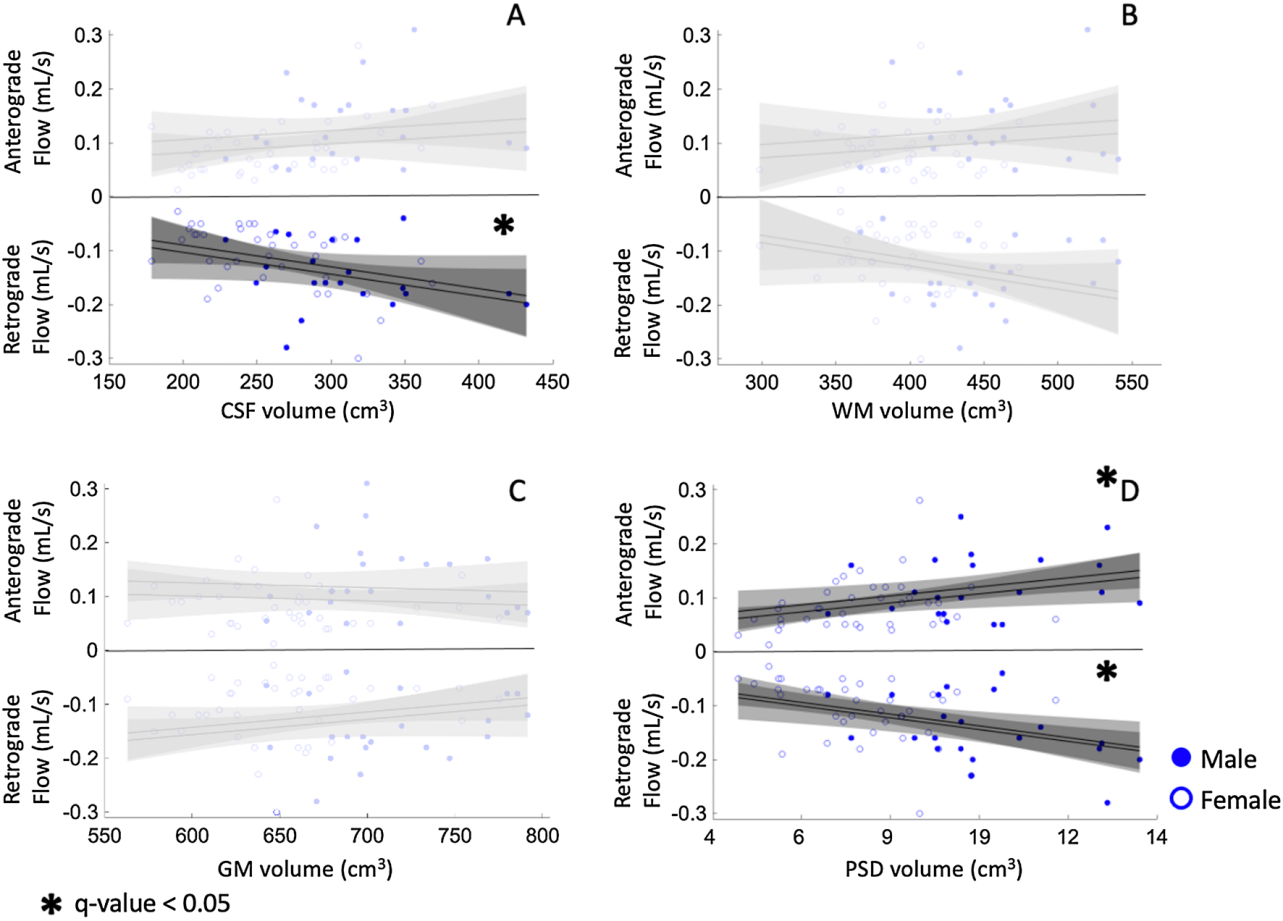
Analysis of relationship between anterograde and retrograde cerebrospinal fluid (CSF) flow in the cerebral aqueduct and CSF, gray matter (GM), white matter (WM), and parasagittal dural (PSD) volumes. The correlation analysis of WM and GM are non-significant. CSF volumes are negatively correlated with retrograde CSF flow (p-value = 0.04, q-value = 0.08). PSD volume correlate with both anterograde and retrograde CSF flow (p-values = 0.01 and 0.001, q-values = 0.04, 0.004). Gray area corresponds to 95 percent confidence intervals estimated using the Wald method. Relationships that do not reach the significant threshold after multiple comparison correction appear in light gray shade; significant relationships are denoted with an asterisk and with confidence intervals appearing in dark gray shade

Figure 2 shows an example of the PSD segmentation process and Fig. 3 demonstrates representative CSF flow data from two volunteers. Figures 4, 5 summarize the relationships between tissue volumes, including PSD volume, and age, whereas Fig. 6 summarizes the relationship between PSD volume and CSF flow. PSD volume was found to have a significant relationship with age: p < 0.001 (q-values = 0.009) and $\uprho$ = 0.59. Like PSD, maximum anterograde and retrograde CSF flow (i.e., as calculated according to Fig. 1) were also observed to have a significant relationship with age: p = 0.010, and p < 0.001 (q-values = 0.030, and 0.002) and $\uprho$ = 0.36, and − 0.40 for PSD volume, anterograde, and retrograde flow, respectively. A significant sex relationship was observed with PSD volume (p-value < 0.001). However, sex difference was not significant in relation to either anterograde or retrograde CSF flow. These findings are consistent with PSD volume being directly related to CSF flow through the aqueduct, as well as being elevated in males versus females.

The investigation of the relationship of PSD volume and CSF flow with ICV are detailed in Additional file 1: Table S2. We observed that ICV and PSD volume are not significantly related when sex is included as a covariate. Moreover, Spearman’s correlation coefficient did not indicate any explanatory characteristic of ICV over the PSD volume ($\uprho$ = 0.1 and 0.2 with p-values = 0.6 and 0.2, for male and female, respectively).

### CSF flow and brain tissue volume

After confirming the correlation of anterograde and retrograde CSF, along with PSD volume, flow with age and sex, we analyzed their relationships with brain tissue volumes. We observed no significant evidence of a relationship between brain tissue volume and either anterograde or retrograde CSF flow. Maximum retrograde CSF flow showed a significant relationship with CSF volume before controlling for multiple hypothesis testing with a p-value of 0.04, but this did not retain significance after FDR correction (q-value = 0.08). Spearman’s rank correlation method indicated a correlation of retrograde CSF flow and CSF volume, with a correlation coefficient $\uprho$ = -0.45 (p-value < 0.001). GM and WM volumes were not significantly related to anterograde or retrograde flow (p = 0.69, 0.42 and 0.14, 0.08 for anterograde and retrograde, respectively) (Figs. 5, 6, Additional file 1: Table S3, S4).

Using the same method, we assessed the relationship between PSD volume and brain tissue and CSF volumes (Figs. 4, 5). No evidence of a correlation between PSD enlargement and GM or WM volume was observed. PSD volume was observed to positively correlate with CSF volume with p-values = 0.02 (q-value = 0.04). In addition, the correlation analysis using Spearman’s correlation coefficient indicated a strong correlation of PSD volume with a correlation coefficient $\uprho$ = 0.6 (p < 0.001).

### Parasagittal dura space and CSF flow characteristics

Two linear models were applied to evaluate the relationship between PSD volume and maximum CSF flow through the cerebral aqueduct. The results provide evidence for a significant correlation of PSD volume and CSF flow for both anterograde and retrograde directionality. The model indicates that anterograde CSF flow correlates with PSD volume with a p-value = 0.01 (q-value = 0.04), and a Spearman’s correlation coefficient of 0.36 (p-value = 0.001). Retrograde CSF flow showed stronger correlation, with a p-value = 0.001 (q-value = 0.004), and a Spearman’s correlation coefficient of $\uprho$ = 0.50 (p-value < 0.001). For completeness, we performed additional supplementary analyses considering net flow volume, absolute flow volume, and regurgitant fraction. Our analysis indicated a positive correlation between regurgitant fraction and PSD volume with p-value inferior to 0.001 (q-value = 0.001) and a correlation coefficient $\uprho$ = 0.33 (p-value = 0.009), see Additional file 1: Figs. S1–S3 and Tables S5–S7.

**Fig. 7:**
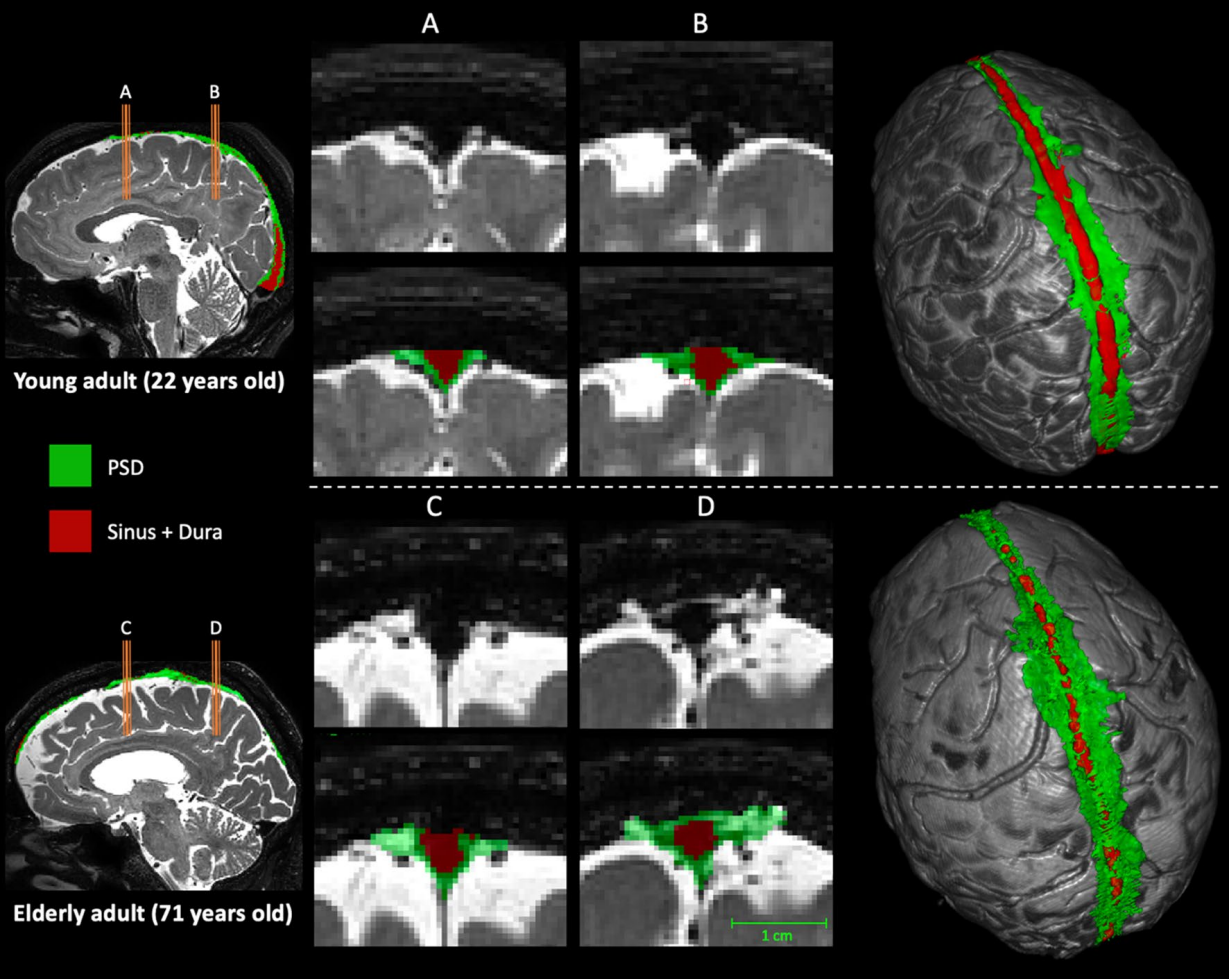
Difference of PSD volume between a young adult (top row) and older adult (bottom row). Label maps are produced by the automatic segmentation method. In red, voxels assigned as sinus and contributing veins, in green voxels assigned as PSD space. The two middle columns show the slices extracted on the: (**A**, **C**) posterior aspect of the frontal lobe, and (**B**, **D**) the anterior aspect of the parietal lobe (i.e., area that shows greatest PSD volume enlargement). On the right, 3D surfaces representation of the brain and its PSD

Figure 7 shows representative cases of a young and older adult, which illustrates the group-level observation of PSD volume increasing with age.

## Discussion

CSF egress pathways along dural sinuses have recently gained attention given their potential importance to cerebral waste clearance, which has in turn prompted a reevaluation of neuro-fluid circulation and relevance. It has recently been suggested that such fluid clearance may occur along parasagittal dural spaces, which co-localize with the anatomic region surrounding the superior sagittal sinus [1] and compelling evidence supports the presence of trans-arachnoid molecular passage in this region [2]. These findings have been shown in concomitance with a study indicating the presence of lymphatic vessels in the PSD space in human and nonhuman primates [1]. However, our understanding of the anatomical and functional relevance of this region remains incomplete, partly due to a lack of robust methods for evaluating this space on neuroimaging, as well as how this space changes with age, sex, and standard measures of bulk CSF flow. Here, we provide evidence that the PSD volume can be assessed non-invasively in vivo using deep learning algorithms applied to high resolution non-contrasted *T*_2_-weighted imaging, and furthermore, that PSD hypertrophy correlates with age and directly relates to CSF flow through the cerebral aqueduct.

### Parasagittal dural volume and potential relevance to CSF egress

It is noteworthy that parasagittal dural space is not the sole potential pathway for CSF egress. Alternative routes have been hypothesized, such as routes involving arachnoid villi and granulations, olfactory pathways through the cribriform plate, and outflow along the spinal canals [18]; these routes may be operative instead of, or in addition to, potential PSD routes that were anatomically evaluated here. Furthermore, it is useful to consider the current findings in the context of other studies that have evaluated CSF egress along PSD spaces with more direct measures of apparent glymphatic flow from tracer-based neuroimaging studies following intrathecal gadolinium administration. In this study we did not measure glymphatic flow, which is difficult to assess directly in humans using available methods, and therefore it is not possible to assess how the findings relate to potential glymphatic changes with aging. However, the proposed glial-lymphatic system, or glymphatic system, has prompted a reassessment of CSF as an important mediator in neurologic waste product clearance.

Increasing animal and human evidence supports the presence of this highly organized system whereby fluid passes from the subarachnoid space to the periarterial space *en route* to the brain parenchymal interstitial space [19–22]. ISF efflux from the brain parenchymal spaces through perivenous spaces has been hypothesized as a pathway for hydrophilic solute fluid movement [23, 24]. Net fluid motion has been hypothesized to exit the interstitial space via the perivenous space en route back to the subarachnoid space, however, owing to limited available methods for characterizing these pathways in vivo, this has not been fully characterized. Impairment of this system has been suggested as a mechanism of waste product accumulation which may underly the development of multiple neurodegenerative diseases with unknown etiology, including but not limited to Alzheimer’s disease, Parkinson’s disease, and multiple sclerosis [25, 26].

The PSD spaces, along with other pathways, have been proposed to help clear fluid exiting the interstitial space. For example, Aspelund et al. demonstrated the importance of a dural lymphatic network for the clearance of macromolecules using transgenic mouse models expressing a VEGF-C/D trap and associated aplasia of dural lymphatics [27]. Here, a reduction in macromolecular transport to deep cervical lymphatic nodes was observed, thereby highlighting the relevance of lymphatic vessels in macromolecular clearance from the mouse CNS. In this work, lymphatic vessels were observed to course downward to the superior sinus and major veins. Presented data suggest that such lymphatic vessels drain CSF and ISF via the foramina of the base of the skull alongside arteries, veins, and cranial nerves to the deep cervical lymphatic nodes. The authors suggest that ISF is cleared from the subarachnoid space along the dura mater lymphatic vessels, which may also propagate along the cranial nerves and cribriform plate. The topography of such channels has not been fully elucidated, and the methods proposed here may help to understand a potential role of the PSD space in ISF egress from the subarachnoid space to potential cervical lymph nodes.

Complementary findings have been observed by Louveau et al. [28]. Using a post-mortem mouse-model and immunohistochemical assays to evaluate the immune cells that occupy the meningeal spaces, evidence was provided supporting lymphatic vessels lining the dural sinuses. Using two fluorescent contrast tracers to differentiate cardiovascular and non-cardiovascular vessels, the experiments suggests that these dural lymphatic vessels drain CSF. This work argues in favor the of the potential relevance of the spaces near the dural sinuses in CSF drainage.

Additionally, in vivo measurements in humans demonstrating trans-arachnoid molecular passage in the PSD region were conducted by introducing gadolinium contrast into the subarachnoid space via intrathecal injection [2], with subsequent *T*_1_-weighted imaging detailing contrast progression to the PSD. Following this finding, a more recent study using intrathecal gadobutrol injection assessed the clearance of CSF in humans over an extended time period of 48 h in adults with CSF leakage disorders [29]. This study observed an increase of the CSF tracer in the blood after approximately 10 h, yet a delayed increase in the tracer in extracranial lymphatic structures, which peaked at approximately 24 h post-injection. Findings from this study suggested that the spinal canal may have a prominent role in CSF clearance. Interestingly, the study also observed a change in CSF clearance related to aging, which could be consistent with the findings in the current study. However, this study noted no large gadolinium drainage from the PSD space, which was suggested and demonstrated using different methodologies in prior work [2].

A separate study assessed so-called peri-sinus lymphatic space volume in 165 neurologically healthy adults using black blood MRI imaging with *T*_1_-weighted postcontrast after gadobutrol injection; participants were undergoing clinically-indicated scanning for suspected or confirmed cancer and brain metastases screening [30]. Consistent with our work, this study observed larger peri-sinus lymphatic spaces in the elderly compared to younger participants (3323 ± 758.7 mL vs. 2968.7 ± 764.3 mL).

In our study, we used submillimeter 3D *T*_2_-weighted imaging of the brain to assess similar volumes without exogenous contrast agent injection. The technique we used allows for distinction of the PSD from both the adjacent subarachnoid space as well as the superior sagittal sinus and feeding cortical veins. Our delineation of this PSD volume is visibly and topographically similar to the 2D appearance and the 3D volumetric maps as detailed by Ringstad et al. [2] using MRI following intrathecal gadobutrol injection. Importantly, the advantage of our method is that it obviates the need for contrast enhanced imaging to determine the PSD volume, which may make it more generalizable for future investigations of CSF egress pathways.

Our results also confirm the previous findings by Park et al. that demonstrated increasing PSD volumes with age [30]. Our study expands upon this work to demonstrate that these increased PSD volumes are associated with increased CSF volume, though is not related to brain parenchymal volume. This observation is significant, as it implies that increased PSD cannot be explained simply by brain volume loss and therefore my provide insight into the pathophysiology of other neurodegenerative processes. Our study benefits from a wider cohort age range compared to this previous work (mean age = 62.1 years, $\sigma$ = 10.9 years, compared to our cohort with mean age = 50.4 years, $\sigma$ = 18 years). We believe that the current cohort which ranged in age from 20 to 83 years enables us to detect evolution of PSD in a more comprehensive manner over the approximate adult human lifespan.

### Cerebral aqueductal flow and parasagittal dural space volume

We observed that PSD volumes were significantly correlated with maximum anterograde and retrograde CSF flow through the cerebral aqueduct. This correlation presents further evidence of an organized system of CSF circulation and metabolism and suggests of complex physiologic interplay between various anatomic structures. Further work is needed across the human lifespan and in disease to determine the sequence of dysfunction across these components and components of the glymphatic circuit. Investigations which characterize these findings across differing neurodegenerative disease cohorts may shed light on how aberrant CSF flow contributes to various neurodegenerative conditions.

We also assessed for a relationship between CSF flow through the cerebral aqueduct with white and gray matter volumes, CSF volume, gender, and age. Our experiments indicate that CSF volumes show significant correlation with maximum retrograde CSF flow in the cerebral aqueduct. This again supports that these findings cannot be fully explained by brain volume loss.

In one prior study investigating the relationship of different CSF dynamics at the level of the cerebral aqueduct, CSF movement was shown to be dependent on age and gender [8]. Only part of these readouts can be confirmed by our experiments, even though we see correlation with age, sex difference was inconclusive in our analysis. However, it is noteworthy that maximum anterograde and retrograde CSF flow have not been directly investigated in this previous study, this could explain why we did not observe gender dependencies with CSF flow in our data.

Finally, while our primary analysis focused on maximum anterograde and retrograde flow through the aqueduct, we performed supplementary analyses where we also considered net flow, absolute flow, and regurgitant fraction (e.g., see Fig. 1). With the exception of regurgitant fraction, these comparisons yielded largely less obvious relationships between PSD volume and CSF flow, consistent with the maximum flow within the cardiac cycle, rather than time-averaged flow properties, being more closely related to PSD volume.

### Technical observations

The imaging and analysis methods proposed enable the assessment of PSD and CSF flow without exogenous agents, however several technical considerations should be noted for successful completion of these methods.

First, for the CSF flow measurements, we used a velocity encoding gradient of 12 cm/s and a slice planned orthogonal to approximately the narrowest region of the cerebral aqueduct. This velocity encoding gradient was chosen as it is near, but greater, than the expected typical CSF flow velocity. However, as observed here the CSF flow becomes higher and more turbulent with increasing age. This can lead to the maximum flow velocity increasing above 12 cm/s, especially in the central voxels of the aqueduct, in older adults. This occurrence leads to phase aliasing, which can be corrected when the directionality of flow is known, which is the case when cardiac monitoring is used and as was performed here. We observed this in five older participants, and without correction, such high flow velocity can lead to potential ambiguous retrograde flow during the time epoch of highest systolic CSF flow. We believe that post-processing correction is a reasonable strategy for quantification, as increasing the velocity encoding gradient beyond 12 cm/s in all participants will make the measurement less accurate for the majority of participants where flow velocity is much lower. However, care should be taken in determining this value and different values based on age and patient population may be worth considering.

Second, in parallel work, we explored the possibility of faster imaging using compressed sensing and lower spatial resolution (e.g., 1 mm isotropic) for the *T*_2_-weighted scans and associated PSD volume deep learning acquisitions. We observed much poorer correspondence with expected regions when a spatial resolution of 1 mm or above (isotropic) were utilized, and also that compressed sensing algorithms led to similarly poorer performance. The proposed non-contrasted scan is 0.8 mm isotropic without compressed sensing and requires a duration of approximately 5 min. We believe that future work to reduce scan times is warranted, however we recommend caution for applying PSD segmentation at lower spatial resolution or with advanced accelerated acquisition or reconstruction algorithms without further refinement.

Finally, extensive work has been conducted to design an effective algorithm for the automatic delineation of PSD space using non-contrasted anatomical MRI. We believe that the benefit of using such techniques is twofold. First, dedicated computer-aided delineation techniques have demonstrated increased accuracy and enables the study of large-scale datasets. Second, and more importantly, these techniques enable us to substantially improve the reproducibility of studies. Therefore, in our work, we adapted recent advances in machine learning with a fully convolutional neural network that has been combined with non-learning statistical modeling of the *T*_2_-weighted signal distribution. Among the many proposed deep-learning architectures, a variant of the well-known U-net architecture showed the best performance in the estimation of the parasagittal mask. Following this initial step, our experiments indicated that techniques based on maximizing a posteriori model using the Bayes framework provide a robust delineation of PSD over superior sinus, afferent veins, and dura mater.

Visual quality assurance done over the entire dataset by our expert radiologist suggest that there is no overestimation of the PSD volume due to size of the sinus. However, it is noteworthy that we did notice some areas appearing as arachnoid granulations can mimic partial occlusion of the superior sinus lumen. This has been reported in a few of case studies in the past [31, 32]. This could be potentially studied using our algorithm in a future work.

### Limitations

The study findings should also be considered in light of several limitations. First, we evaluated the PSD volume across the lifespan cross-sectionally as is common in neuroimaging studies, rather than longitudinally. Second, while the largest cohort of PSD volume data presented to date, the sample size of 62 was moderate and precluded multiple co-variates from being included in analysis. However, we characterized the health of each participant both radiologically and neurologically as described in the inclusion criteria, and all participants met rigorous healthy volunteer criteria. Given the moderate sample size, we also focused hypotheses on those that could be tested responsibly with the sample size.

## Conclusions

Findings highlight the feasibility of quantifying PSD volume non-invasively in vivo in humans using deep learning and non-contrasted submillimeter *T*_2_-weighted MRI, that PSD volume increases with age, and that PSD volume relates to bulk CSF volume and maximum bi-directional flow through the cerebral aqueduct. Values reported should provide useful normative ranges for how PSD volume adjusts with age, which will serve as a necessary pre-requisite for comparisons to persons with neurodegenerative disorders. Findings also provide new tools that may help to complement the growing number of studies of CSF egress. These processes may contribute to age-related neurological changes and possible vulnerability to further pathological disruption of CSF clearance processes.

## Supplementary Information

**Additional file 1.** Coefficients of regression analyses and results for the analysis of the relationship of additional CSF flow measures (i.e., net flow, absolute flow, and regurgitant fraction) and anatomical measures (i.e., GM, WM, CSF, and PSD volumes).

## Data Availability

The data that support the findings of this study are available from the corresponding author, MJD, upon reasonable request.
