# Supplementary material for: Parasagittal dural space and cerebrospinal fluid (CSF) flow across the lifespan in healthy adults
Source: Fluids Barriers CNS. 2022 Mar 21;19:24. doi: 10.1186/s12987-022-00320-4 (PMC8935696; doi:10.1186/s12987-022-00320-4)
Supplement: Supplementary file 1 — Additional file 1. Coefficients of regression analyses and results for the analysis of the relationship of additional CSF flow measures (i.e., net flow, absolute flow, and regurgitant fraction) and anatomical measures (i.e., GM, WM, CSF, and PSD volumes). [file 12987_2022_320_MOESM1_ESM.docx]

## **Parasagittal dural space and cerebrospinal fluid (CSF) flow across the lifespan in healthy adults**

Kilian Hett^1^, Colin D. McKnight^2^, Jarrod J. Eisma^1^, Jason Elenberger^1^, Jennifer S. Lindsey^2^, Ciaran M. Considine^1^, Daniel O. Claassen^1^, and Manus J. Donahue^1,3*^

^1^ Department of Neurology, Vanderbilt University Medical Center, Nashville, TN, USA

^2^ Department of Radiology and Radiological Sciences, Vanderbilt University Medical Center, Nashville, TN, USA

^3^ Department of Psychiatry and Behavioral Sciences, Vanderbilt University Medical Center, Nashville, TN, USA

**Supplementary table count:** 7

**Supplementary figure count:** 3

SUPPLEMENTARY MATERIALS

*** Corresponding author**

Manus J. Donahue, PhD

1500 21^st^ Avenue South

Village at Vanderbilt

Division of Cognitive Disorders

Nashville, TN, 37069

Tel: 615.939.2869

Email: mj.donahue@vumc.org

| Variables | $\boldsymbol{\beta}$ coefficient (Std err.) | p-value (q-value FDR) | $\boldsymbol{\rho}$ score (p-value) |
| --- | --- | --- | --- |
| CSF | **1.76 (0.25)** | **6.4e-12 (1.3e-11)** | **0.79 (1.2e-14)** |
| GM | **-1.24 (0.35)** | **6.3e-13 (2.5e-12)** | **-0.81 (1.4e-15)** |
| WM | -0.34 (0.32) | 0.29 (0.29) | -0.08 (0.54) |
| Retrograde flow | **9.1e-4 (3.6e-4)** | **0.01 (0.03)** | **0.36 (5.7e-03)** |
| Anterograde flow | **-0.001 (3.8e-4)** | **9.0e-04 (2.7e-03)** | **-0.40 (1.9e-03)** |
| Net flow | **9.1e-04 (2.5e-4)** | **5.0e-04 (2.4e-03)** | **0.44 (4.8e-04)** |
| Regurgitant Fraction | **0.008 (0.001)** | **4.4e-05 (2.0e-03)** | **0.60 (4.1e-07)** |
| PSD | **0.003 (0.001)** | **7.0e-03 (9.0e-03)** | **0.59 (7.2e-07)** |

***Table A1****. Statistics of relationship analysis with age and different anatomical variables (CSF, GM, anterograde or retrograde maximum flow, net flow, regurgitant fraction, and PSD volumes).* $\boldsymbol{\beta}$ *coefficients were estimated using multivariate GLM including age and sex as covariates.* $\rho$ *correlation scores have been estimated using the Spearman’s rank correlation method. The results indicate that age correlates with CSF, GM, WM, which have been previously shown in many studies, and PSD, which is being observed for the first time. In this table, significant relationships appears in bold and relationships with p-value < 0.05 but which do not survive FDR correction are underlined.*

| Variables | $\boldsymbol{\beta}$ coefficient (std err.) | p-value (q-value FDR) | $\boldsymbol{\rho}$ coef. (p-value) |
| --- | --- | --- | --- |
| ICV _male_ | 0.005 (0.005) | 0.29 (0.68) | 0.10 (0.6) |
| ICV _female_ | 0.002 (0.003) | 0.51 (1.00) | 0.20 (0.2) |
| CSF | **0.01 (0.004)** | **0.02 (0.04)** | **0.60 (5.3e-07)** |
| GM | 0.009 (0.006) | 0.11 (0.34) | 0.29 (0.02) |
| WM | -0.007 (0.006) | 0.24 (0.34) | 0.34 (0.007) |

***Table A2.*** *Statistics of relationship analysis of PSD volumes with different anatomical, and demographic variables (ICV, CSF, GM, WM volumes, and sex).* $\boldsymbol{\beta}$ *coefficients were estimated using multivariate GLM including age and sex as covariates.* $\rho$ *correlation coefficients were computed using Spearman’s rank correlation method. In this table, significant relationships appear in bold and relationships with p-value < 0.05 but which do not survive FDR correction are underlined.*

| Variables | $\boldsymbol{\beta}$ coefficient (std err.) | p-value (q-value FDR) | $\boldsymbol{\rho}$ coef. (p-value) |
| --- | --- | --- | --- |
| ICV | 1.7e-5 (3.4e-5) | 0.60 (0.63) | 0.28 (3.4e-2) |
| CSF | 0.0002 (0.0002) | 0.32 (0.56) | 0.31 (0.02) |
| GM | -0.0001 (-0.0003) | 0.69 (0.69) | 0.11 (0.40) |
| WM | 0.0002 (0.0003) | 0.42 (0.56) | 0.19 (0.15) |
| PSD | **0.008 (0.003)** | **0.01 (0.04)** | **0.36 (0.002)** |

***Table A3.*** *Statistics of relationship analysis with the maximum anterograde CSF flow (i.e., movement of CSF through the aqueduct of Sylvius) and different anatomical variables (ICV, CSF, GM, and PSD volumes).* $\boldsymbol{\beta}$ *coefficients are estimated using multivariate GLM including age and sex as covariates.* $\rho$ *correlation scores have been estimated using Spearman’s rank correlation method. The results indicate that age correlates with CSF, GM, WM, which has been previously shown in many studies, and PSD, which is being observed for the first time in healthy aging. In this table, significant relationships appears in bold.*

| Variables | $\boldsymbol{\beta}$ coefficient (std err.) | p-value (q-value FDR) | $\boldsymbol{\rho}$ coef. (p-value) |
| --- | --- | --- | --- |
| ICV | -2.3e- 5 (3.6e-5) | 0.51 (0.66) | -0.36 (6.0e-3) |
| CSF | -0.0004 (0.0002) | 0.04 (0.08) | -0.45 (4.1e-4) |
| ­GM | 0.0004 (0.0003) | 0.14 (0.14) | -0.08 (0.56) |
| WM | -0.0005 (0.0003) | 0.08 (0.10) | 0.19 (0.15) |
| PSD | **-0.01 (0.003)** | **0.001 (0.004)** | **-0.50 (7.3e-5)** |

***Table A4.*** *Statistics of relationship analysis with maximum retrograde CSF flow (i.e., in the aqueduct of Sylvius) and different anatomical variables (ICV, CSF, GM, and PSD volumes).* $\boldsymbol{\beta}$ *coefficients are estimated using multivariate GLM including age and sex as covariates.* $\rho$ *correlation scores have been estimated using Spearman’s rank correlation method. In this table, significant relationships appear in bold.*

| Variables | $\boldsymbol{\beta}$ coefficient (std err.) | p-value (q-value FDR) | $\boldsymbol{\rho}$ coef. (p-value) |
| --- | --- | --- | --- |
| ICV | -3.4e-06 (3.9e-06) | 0.38 (0.66) | 0.30 (0.16) |
| CSF | 7.2e-06 (1.1e-05) | 0.54 (0.54) | 0.13 (0.34) |
| ­GM | -1.6e-05 (9.6e-06) | 0.09 (0.42) | -0.38 (3.3e-3) |
| WM | 9.7e-06 (1.5e-05) | 0.52 (0.54) | -0.25 (0.05) |
| PSD | -4.6e-04 (3.0e-04) | 0.13 (0.22) | -0.07 (0.62) |

***Table A5.*** *Statistics of relationship analysis with net flow volume (i.e., total movement of CSF) in the aqueduct of Sylvius and different anatomical variables (ICV, CSF, GM, and PSD volumes).* $\beta$ *coefficients are estimated using multivariate GLM including age and sex as covariates.* $\rho$ *correlation scores have been estimated using Spearman’s rank correlation method. In this table, significant relationship appears in bold.*

| Variables | $\boldsymbol{\beta}$ coefficient (std err.) | p-value (q-value FDR) | $\boldsymbol{\rho}$ coef. (p-value) |
| --- | --- | --- | --- |
| ICV | 4.1e-05 (5.3e-05) | 0.44 (0.70) | 0.14 (0.26) |
| CSF | 9.2e-05 (0.0001) | 0.55 (0.55) | 0.51 (2.5e-5) |
| ­GM | -0.0001 (0.0001) | 0.12 (0.15) | 0.09 (0.49) |
| WM | 0.0003 (0.0001) | 0.55 (0.55) | 0.22 (0.08) |
| PSD | 0.003 (0.002) | 0.14 (0.14) | 0.45 (3.5e-4) |

***Table A6.*** *Statistics of relationship analysis with absolute flow volume (i.e., total movement of CSF) in the aqueduct of Sylvius and different anatomical variables (ICV, CSF, GM, and PSD volumes).* $\beta$ *coefficients are estimated using multivariate GLM including age and sex as covariates.* $\rho$ *correlation scores have been estimated using Spearman’s rank correlation method. In this table, significant relationships appear in bold.*

| Variables | $\boldsymbol{\beta}$ coefficient (std err.) | p-value (q-value FDR) | $\boldsymbol{\rho}$ coef. (p-value) |
| --- | --- | --- | --- |
| ICV | 0.0001 (0.0003) | 0.74 (0.74) | -0.8 (0.51) |
| CSF | 0.0007 (0.001) | 0.45 (0.46) | 0.51 (3.3e-5) |
| ­GM | 0.001 (0.0008) | 0.07 (0.18) | -0.17 (0.18) |
| WM | -0.001 (0.001) | 0.20 (0.34) | 0.00 (0.99) |
| PSD | **0.06 (0.01)** | **7.9e-4 (0.001)** | **0.33 (9.5e-3)** |

***Table A7.*** *Statistics of relationship analysis with regurgitant fraction of CSF (i.e., ratio of CSF volume) in the aqueduct of Sylvius and different anatomical variables (ICV, CSF, GM, and PSD volumes).* $\beta$ *coefficients are estimated using multivariate GLM including age and sex as covariates.* $\rho$ *correlation scores have been estimated using Spearman’s rank correlation method. In this table, significant relationships appear in bold.*

**
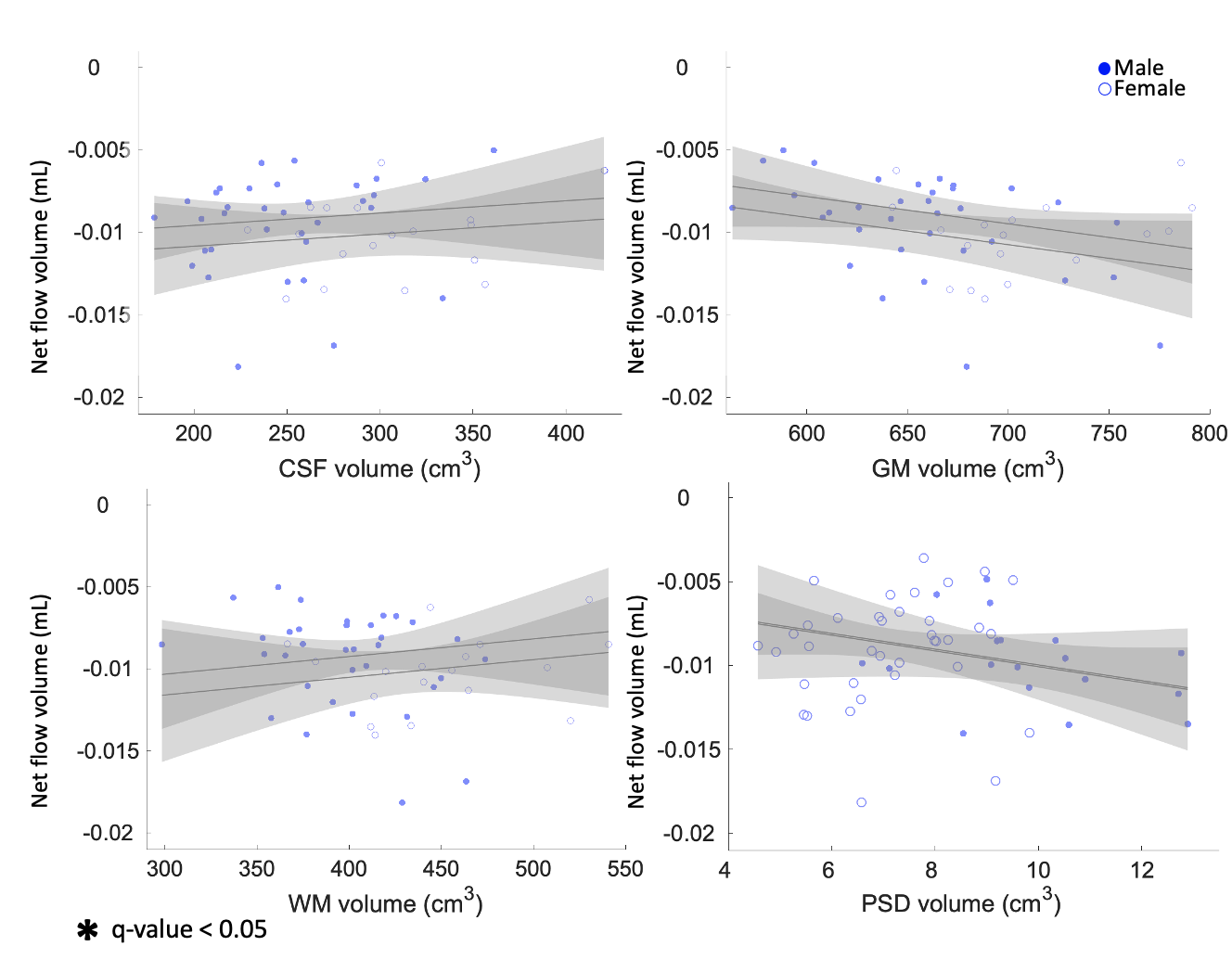
**

**Figure A1.** Analysis of relationship between net flow volume of cerebrospinal fluid (CSF) flux in the cerebral aqueduct and CSF, gray matter (GM), white matter (WM), and parasagittal dural (PSD) volumes. The correlation analysis of WM and GM are non-significant. CSF volumes are negatively correlated with retrograde CSF flow (p-value = 0.04, q-value = 0.08). PSD volume correlates with both anterograde and retrograde CSF flow (p-values = 0.01 and 0.001, q-values = 0.04, 0.004). Gray area corresponds to 95 percent confidence intervals estimated using the Wald method. Relationships that do not reach the significant threshold after multiple comparison correction appear in light gray shade; significant relationships are denoted with an asterisk and with confidence intervals appearing in dark gray shade.

**
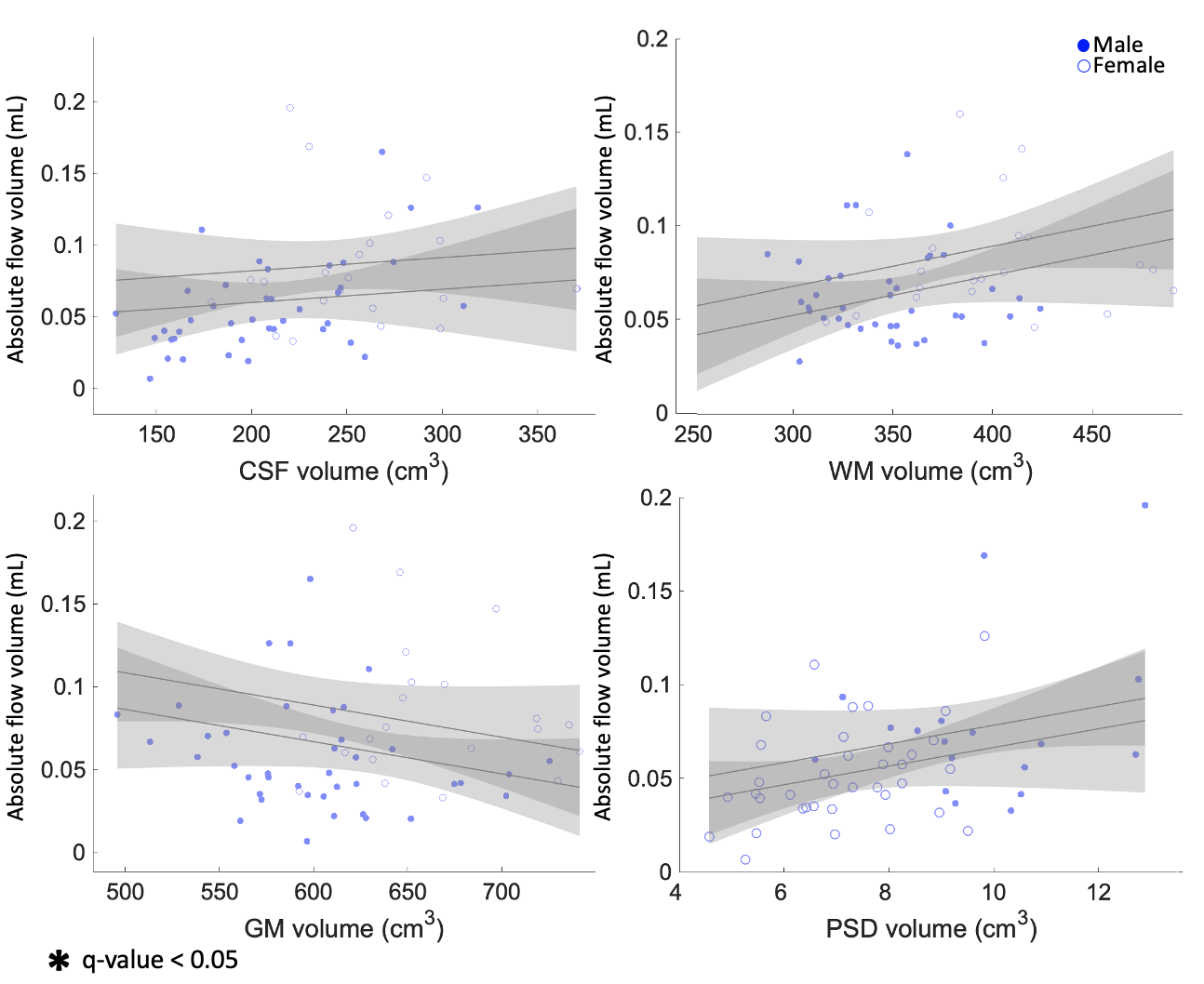
**

**Figure A2.** Analysis of relationship between absolute flow volume of cerebrospinal fluid (CSF) flow in the cerebral aqueduct and CSF, gray matter (GM), white matter (WM), and parasagittal dural (PSD) volumes. The correlation analysis of WM and GM are non-significant. CSF volumes are negatively correlated with retrograde CSF flux (p-value = 0.04, q-value = 0.08). PSD volume correlate with both anterograde and retrograde CSF flow (p-values = 0.01 and 0.001, q-values = 0.04, 0.004). Gray area corresponds to 95 percent confidence intervals estimated using the Wald method. Relationships that do not reach the significant threshold after multiple comparison correction appear in light gray shade; significant relationships are denoted with an asterisk and with confidence intervals appearing in dark gray shade.

**
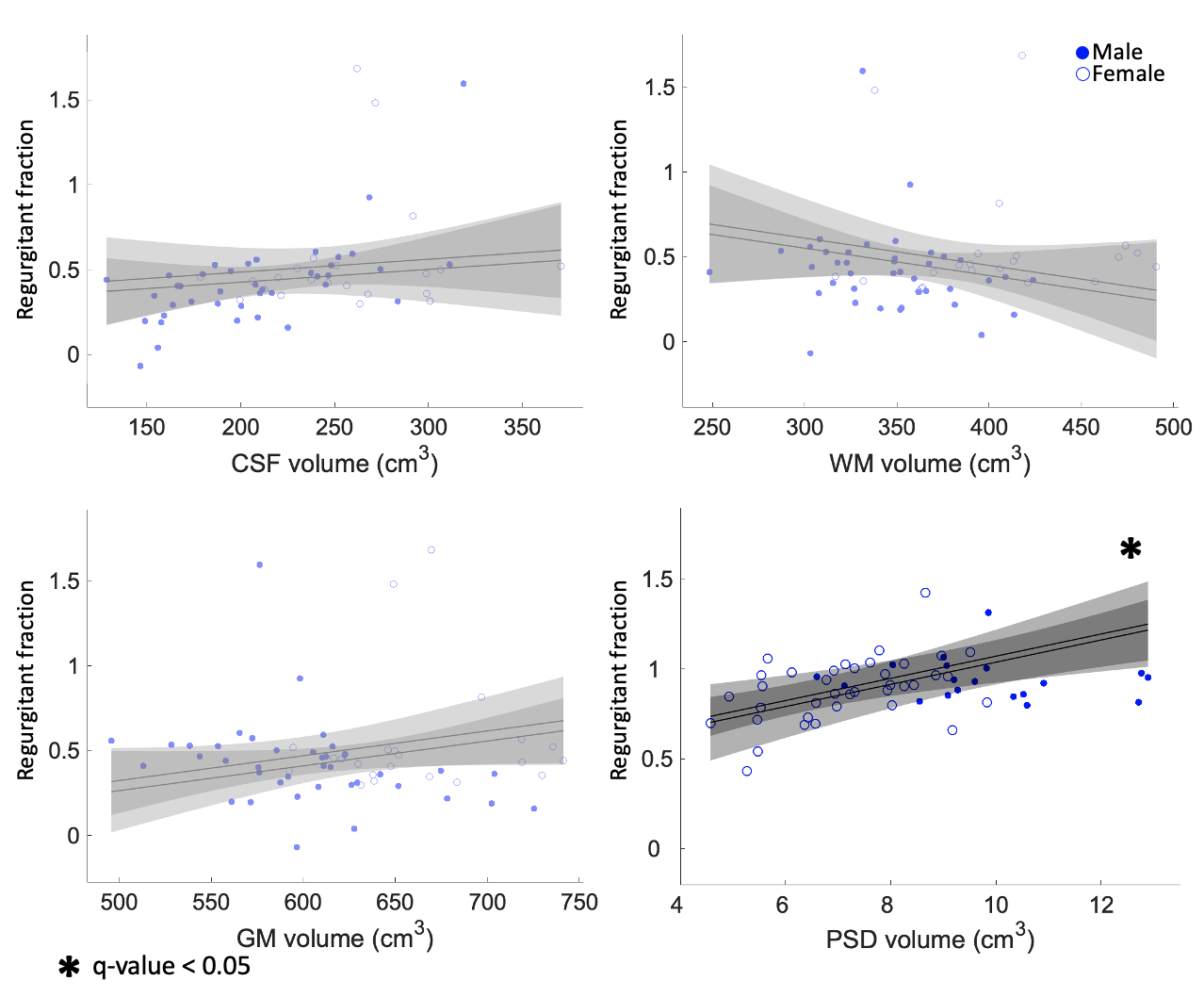
**

**Figure A3.** Analysis of relationship between regurgitant fraction of cerebrospinal fluid (CSF) flow in the cerebral aqueduct and CSF, gray matter (GM), white matter (WM), and parasagittal dural (PSD) volumes. The correlation analysis of WM and GM are non-significant. CSF volumes are negatively correlated with retrograde CSF flow (p-value = 0.04, q-value = 0.08). PSD volume correlates with both anterograde and retrograde CSF flow (p-values = 0.01 and 0.001, q-values = 0.04, 0.004). Gray area corresponds to 95 percent confidence intervals estimated using the Wald method. Relationships that do not reach the significant threshold after multiple comparison correction appear in light gray shade; significant relationships are denoted with an asterisk and with confidence intervals appearing in dark gray shade.
